# Supplementary material for: On the importance of the electric double layer structure in aqueous electrocatalysis
Source: Nat Commun. 2022 Jan 10;13:174. doi: 10.1038/s41467-021-27909-x (PMC8748683; doi:10.1038/s41467-021-27909-x)
Supplement: Supplementary file 1 — Supplementary Information [file 41467_2021_27909_MOESM1_ESM.pdf]

Supplementary Information for

# On the importance of the electric double layer structure in aqueous electrocatalysis

**AUTHORS:** Seung-Jae Shin[1,†], Dong Hyun Kim[2,†], Geunsu Bae[2,†], Stefan Ringe[3,4], Hansol Choi[2], Hyung-Kyu Lim[5], Chang Hyuck Choi[2,*], and Hyungjun Kim[1,*].

**Affiliations:**

[1]Department of Chemistry, Korea Advanced Institute of Science and Technology, Daejeon 34141, Republic of Korea.

[2]School of Materials Science and Engineering, Gwangju Institute of Science and Technology, Gwangju 61005, Republic of Korea.

[3]Department of Energy Science and Engineering, Daegu Gyeongbuk Institute of Science and Technology, Daegu 42988, Republic of Korea.

[4]Energy Science and Engineering Research Center, Daegu Gyeongbuk Institute of Science and Technology (DGIST), Daegu 42988, Republic of Korea

[5]Division of Chemical Engineering and Bioengineering, Kangwon National University, Chuncheon, Gangwon-do 24341, Republic of Korea.

†These authors contributed equally to this work.

Correspondence to: chchoi@gist.ac.kr (C.H.C.); linus16@kaist.ac.kr (H.K.)

**This PDF file includes:**

Supplementary Note 1 to 6

Supplementary Fig. 1 to 12

Supplementary References

## Supplementary Notes

Supplementary Note #1. Computational details

The Ag(111) electrode was quantum-mechanically modeled using a four-layer slab with a $(\sqrt{3} \times 2)$rect surface unit cell with the dimensions of 5.08 Å × 5.86 Å. The projector-augmented-wave (PAW)[1] method was used with a kinetic energy cutoff of 50 Ry. Gaussian smearing was used with a value of 0.2 eV and the Perdew–Burke–Ernzerhof (PBE) exchange-correlation functional was employed[2]. A (8 × 8 × 1) Γ-centered k-point grid was used to sample the reciprocal space, and a dipole correction along the $z$-direction was applied to block the unphysical interaction between the images of the cells.

The electrolyte phase was classically modeled using the canonical ensemble molecular dynamics (MD). 2000 TIP3P-EW[3] water molecules were simulated, and the excess $Na^+$ or $F^-$ ions were included in the molecular mechanics (MM) region to compensate for the excess negative or positive charge of the electrode in the quantum mechanics (QM) region, respectively. A Nosé–Hoover thermostat[4,5] was employed to maintain the temperature at 300 K, with a damping parameter of 100 fs. Periodic boundary conditions (PBCs) were applied along the $x$- and $y$-direction, and the long-range electrostatic interactions in the simulation cells were treated employing the modified particle-particle particle-mesh (PPPM) method for slab geometry[6]. The external potential from the electrode in the QM region was set as follows: The density functional theory (DFT)-optimized structure and electrostatic potential obtained from the $(\sqrt{3} \times 2)$rect surface unit cell was repeated to fill the $(9\sqrt{3} \times 16)$rect surface unit cell, resulting in an MD simulation cell dimension of 45.7 Å × 46.9 Å × 65.0 Å. For the DFT calculation of $CO_2$ adsorbed system, $(2\sqrt{3} \times 4)$rect surface unit cell of Ag(111) with an excess charge of $-1e$ was

used, where one $CO_2$ molecule was adsorbed as a bent form. Then, the QM simulation cell was repeated to fill $(6\sqrt{3} \times 12)$rect unit cell for the MD simulation cell dimension of 30.5 Å × 35.2 Å × 65.0 Å, where 2000 water molecules with excess $Na^+$ ions to compensate the electrode charge were included. Details of the force-field (FF) setting and the first-principles-based FF development strategies are summarized in the **Supplementary Note 4**.

At every density functional theory in classical explicit solvents (DFT-CES) iteration, we performed an MD simulation for 6 ns and sampled the last 5 ns trajectory to calculate the average electrostatic potential of the electrolyte phase that was employed in the subsequent DFT calculation as an external potential. During DFT calculations, only the electronic structure was optimized based on the external potential obtained from the previous MD iteration. The DFT-CES iteration was repeated until the difference of the DFT total energy between the iterations converged below 0.1 kcal $mol^{-1}$. In most cases, the total energy converged within three DFT-CES iterations. The theoretical background and simulation flow of the DFT-CES can be found in the **Supplementary Notes 2 and 3**.

Supplementary Note #2. Theoretical background of mean-field QM/MM

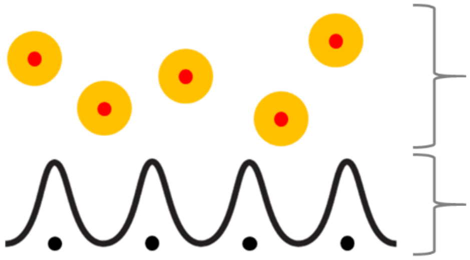


By partitioning the entire system into an QM region (consisting of $N$ atoms) and a MM region (consisting of $N'$ number of atoms), the entire QM/MM Hamiltonian consists of three terms: $H_{\mathrm{QM}}$, $H_{\mathrm{MM}}$, and $H_{\mathrm{Int}}$. Here, the QM system is described based on the Kohn–Sham (KS) DFT under the Born–Oppenheimer approximation, and the interaction potential within the MM system is described based on classical FF.

$$H_{\mathrm{QM/MM}} = H_{\mathrm{QM}} + H_{\mathrm{MM}} + H_{\mathrm{Int}},$$

$$H_{\mathrm{QM}} = \sum_\mu \frac{\mathbf{P}_\mu^2}{2M_\mu} + \min_\rho E^{\mathrm{KS}}[\rho; \{\mathbf{R}_\mu\}],$$

$$H_{\mathrm{MM}} = \sum_i \frac{\mathbf{p}_i^2}{2m_i} + U^{\mathrm{FF}}(\{\mathbf{r}_i\}),$$

where the Greek and Latin letters denote the particles in the QM and MM regions, respectively. In addition, $\mathbf{P}$ ($\mathbf{p}$), $\mathbf{R}$ ($\mathbf{r}$), and $M$ ($m$) denote the momentum, position, and mass of the QM (MM) particles, respectively, and $E^{\mathrm{KS}}[\rho]$ is the KS-DFT energy functional.

Suppose that no chemical bond exists at the boundary of the QM and MM regions, the interaction Hamiltonian between two regions, $H_{\mathrm{Int}}$, is basically defined as a sum of the electrostatic interaction term and van der Waals (vdW) interaction term and is usually expressed as,

$$H_{\text{Int}} = \int V_{\text{MM}} \rho d\mathbf{x}^3 + \sum_\mu \int V_{\text{MM}} Q_\mu \delta(\mathbf{x} - \mathbf{R}_\mu)\, d\mathbf{x}^3 + U^{\text{vdW}}(\{\mathbf{r}_i\}, \{\mathbf{R}_\mu\}),$$

where the first and second terms denote the MM charge–QM electron and MM charge–QM nuclei electrostatic interactions, respectively ($V_{\text{MM}}$ is the electrostatic potential generated by the MM point charges, $\{q_i\}$). The last term refers to the vdW interaction between the QM and MM regions that can be appropriately described based on the atom-centered pairwise potential, $U^{\text{vdW}}$.

When one derives the equations of motion using the above-defined $H_{\text{QM/MM}}$ and numerically processes the propagation of the time trajectory, the simulation becomes a conventional QM/MM simulation based on an electrostatic embedding scheme. However, despite integrating the coarse-grained MM-level description of the electrolyte phase, it requires an impractically high computational cost to time-propagate the dynamics of the metal electrode with a sufficiently large surface area (1 μC cm$^{-2}$ is converted to $1e$ over 1600 Å$^2$, consisting of ~220 atoms only for the topmost lattice points) for a substantially long time duration (an order of nanoseconds, for sufficient equilibrium sampling).

By employing a mean-field approximation to treat the interaction between QM and MM regions, we can decouple the time-scales of QM and MM dynamics and significantly expedite the simulation. In addition, this is a reasonable approach for the solid-liquid interfacial systems when there is no significant vibrational coupling between the solid and liquid molecules. Therefore, we now define a canonical partition function for the entire QM/MM system using $H_{\text{QM/MM}}$. Subsequently, by considering the temporal dynamics of nuclei positions, $\{\mathbf{R}_\mu\}$ and $\rho$ of the QM system as a chain of states, the Helmholtz free energy functional can be derived as follows:

$$Q_{\mathrm{QM/MM}} = \int e^{-\beta H_{\mathrm{QM/MM}}} d\mathbf{r}^{3N'} d\mathbf{p}^{3N'} d\mathbf{R}^{3N} d\mathbf{P}^{3N},$$

$$A_{\mathrm{QM/MM}}[\rho, \{\mathbf{R}_\mu\}] = A_0 - k_B T \ln\langle e^{-\beta H_{\mathrm{QM/MM}}} \rangle_{\{\mathbf{r}_i\},\{\mathbf{p}_i\}},$$

where the bracket $\langle \dots \rangle_{\{\mathbf{r}_i\},\{\mathbf{p}_i\}}$ denotes the ensemble average over the phase space spanned by the MM particle dynamics. Finally, we obtain a variation theory wherein $A_{\mathrm{QM/MM}}$ is minimized in response to the change in $\mathbf{R}_\mu$ (structural relaxation) and $\rho$ (electronic relaxation):

$$\nabla_\mu A_{\mathrm{QM/MM}} = \langle \nabla_\mu H_{\mathrm{QM/MM}} \rangle_{\{\boldsymbol{r}_i\},\{\boldsymbol{p}_i\}} = -\mathbf{F}_\mu^{\mathrm{HF}} + Q_\mu \nabla \overline{V_{\mathrm{MM}}} - \langle \mathbf{F}_\mu^{\mathrm{vdW}} \rangle_{\{\mathbf{r}_i\},\{\mathbf{p}_i\}},$$

$$\frac{\partial A_{\mathrm{QM/MM}}}{\partial \rho} = \langle \frac{\partial H_{\mathrm{QM/MM}}}{\partial \rho} \rangle_{\{\mathbf{r}_i\},\{\mathbf{p}_i\}} = \varepsilon^{\mathrm{KS}}[\rho; \{\mathbf{R}_\mu\}] + \overline{V_{\mathrm{MM}}},$$

where $\mathbf{F}_\mu^{\mathrm{HF}}$ is the Hellmann-Feynman force on the $\mu$'th nucleus, $\mathbf{F}_\mu^{\mathrm{vdW}} = -\sum_i \nabla_\mu U^{\mathrm{vdW}}$, and $\varepsilon^{\mathrm{KS}}$ is the Kohn–Sham potential. The ensemble-averaged electrostatic potential of the MM point charge, $\overline{V_{\mathrm{MM}}} = \langle V_{\mathrm{MM}} \rangle_{\{\mathbf{r}_i\},\{\mathbf{p}_i\}}$, can be obtained based on a classical MD simulation. In this study, we note that the structural relaxation of the electrode part in the QM region was carried out only at the initial DFT calculation in vacuum, while the electron density was fully relaxed at each DFT-CES iteration.

Supplementary Note #3. Grid-based mean-field QM/MM: DFT-CES

Using a real-space grid to interconnect the electrostatic interactions between QM and MM regions, we developed a fully consistent mean-field QM/MM method by combining a planewave DFT simulation and classical MD simulation.

Detailed procedure of the DFT-CES simulation is as follows:

(1) Periodic DFT calculations is performed using the planewave basis set, and the optimized $\rho$ and the total electrostatic potential generated by the QM particles, $V_{\mathrm{QM}}$, are stored in a three-dimensional (3D) grid. The gradient of the electrostatic potential, that is, the electrostatic field, is calculated using the finite difference method (FDM). Here, we obtain the DFT total energy, $E_{\mathrm{total}}$.

(2) Considering the $\{\mathbf{R}_{\mu}\}$ and $\rho$ of the QM particles obtained from the DFT calculations to be invariable, MD simulation was performed for the MM particles. During the MD run, the classical MD particles experience an acceleration force, owing to the electrostatic interaction between the point charge of the MM particle and the external electrostatic field generated by the QM particles (that are stored in the 3D grid transferred from the preceding DFT iteration). Meanwhile, the ensemble average is calculated over the MM point-charge dynamics that is stored in another 3D grid, denoted as $\rho_{\mathrm{MM}}$.

(3) The Poisson equation is solved to obtain $\overline{V_{\mathrm{MM}}}$ that is thereafter transferred to the next DFT calculation. During the subsequent DFT calculation, $\overline{V_{\mathrm{MM}}}$ is applied as an external electrostatic potential to obtain a newly optimized $\rho$ and the total electrostatic potential generated by the QM particles. Thereafter, the MD and DFT simulations are repeated until a self-consistent solution is obtained.

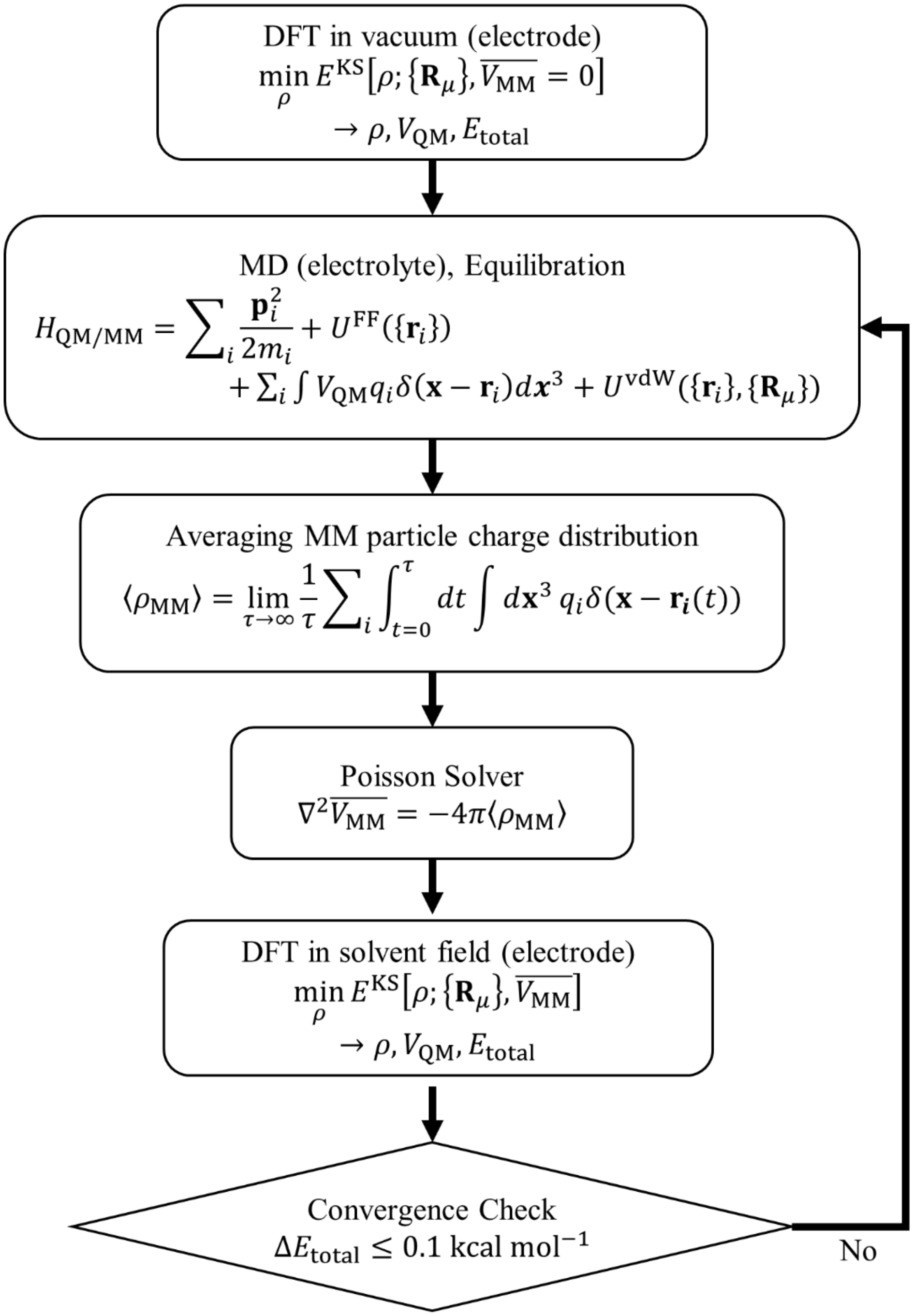
DFT in vacuum (electrode)
$\min_{\rho} E^{\mathrm{KS}}[\rho; \{\mathbf{R}_\mu\}, \overline{V_{\mathrm{MM}}} = 0]$
$\rightarrow \rho, V_{\mathrm{QM}}, E_{\mathrm{total}}$
MD (electrolyte), Equilibration
$H_{\mathrm{QM/MM}} = \sum_i \frac{\mathbf{p}_i^2}{2m_i} + U^{\mathrm{FF}}(\{\mathbf{r}_i\}) + \sum_i \int V_{\mathrm{QM}} q_i \delta(\mathbf{x} - \mathbf{r}_i) d\boldsymbol{x}^3 + U^{\mathrm{vdW}}(\{\mathbf{r}_i\}, \{\mathbf{R}_\mu\})$
Averaging MM particle charge distribution
$\langle \rho_{\mathrm{MM}} \rangle = \lim_{\tau \to \infty} \frac{1}{\tau} \sum_i \int_{t=0}^{\tau} dt \int d\mathbf{x}^3 \, q_i \delta(\mathbf{x} - \mathbf{r}_{\boldsymbol{i}}(t))$
Poisson Solver
$\nabla^2 \overline{V_{\mathrm{MM}}} = -4\pi \langle \rho_{\mathrm{MM}} \rangle$
DFT in solvent field (electrode)
$\min_{\rho} E^{\mathrm{KS}}[\rho; \{\mathbf{R}_\mu\}, \overline{V_{\mathrm{MM}}}]$
$\rightarrow \rho, V_{\mathrm{QM}}, E_{\mathrm{total}}$
Convergence Check
$\Delta E_{\mathrm{total}} \leq 0.1 \text{ kcal mol}^{-1}$
No

Supplementary Note #4. FF setting and first-principles-based development of parameters

For the DFT-CES simulation, we require two sets of classical FF parameters: one describes the interaction among the MM particles ($U^{FF}(\{\mathbf{r}_i\})$), and the other describes the vdW interaction across the boundary between the QM and MM regions ($U^{\mathrm{vdW}}(\{\mathbf{r}_i\},\{\mathbf{R}_\mu\})$). Our strategy is to employ predeveloped FF sets for defining $U^{FF}(\{\mathbf{r}_i\})$ and to develop $U^{\mathrm{vdW}}(\{\mathbf{r}_i\},\{\mathbf{R}_\mu\})$ parameters based on first-principles energetics. This is not only due to the existence of carefully developed FF parameters that can reliably predict the thermodynamics, structure, and dynamics of the liquid electrolyte, but also because of the importance of the interfacial interaction between the electrode (in the QM region) and the electrolyte (in the MM region) that is based on QM energetics.

Water molecules are modeled based on a TIP3P-EW model[3] that reproduces the experimental dielectric constant of ~80, and the ion-water interactions were described using the parameters presented in the reference[7], that accurately predict the hydration free energy of $Na^+$ as 80–90 kcal mol$^{-1}$ (experimental value[8] is 87.2 kcal mol$^{-1}$) and that of $F^-$ as 115–120 kcal mol (experimental value[8] is 111.1 kcal mol$^{-1}$). 15-Crown-5 (15C5) is modeled using DREIDING 2.21 FF[9] and the $CO_2$ adsorbate is modeled using OPLS-AA FF[10].

To carefully develop the FF parameters for an accurate description of the interfacial interaction, we obtain the binding energy curves of a single water molecule and a 15C5 molecule to the Ag(111) surface, employing the vdW-corrected DFT method. We employed a vdW-DF2 functional[11] that is a nonlocal exchange-correlation functional that accurately describes the long-range dispersion correlation energy. We subsequently developed pairwise interaction parameters to reproduce the QM-level Ag–water interaction and the Ag–15C5 interaction. To appropriately

model the soft exchange repulsion of the metal atom with a diffuse electron density, we employed the Buckingham potential with three adjustable parameters: $A_{ij}$, $R_{ij}$, and $C_{6,ij}$. Optimized parameters for Ag–$H_{water}$ and Ag–$O_{water}$ interactions are listed in below table.

$$U_{ij}^{\mathrm{vdW}}(r) = A_{ij}\exp\left(-\frac{r_{ij}}{R_{ij}}\right) - \frac{C_{6,ij}}{r_{ij}^{6}}$$

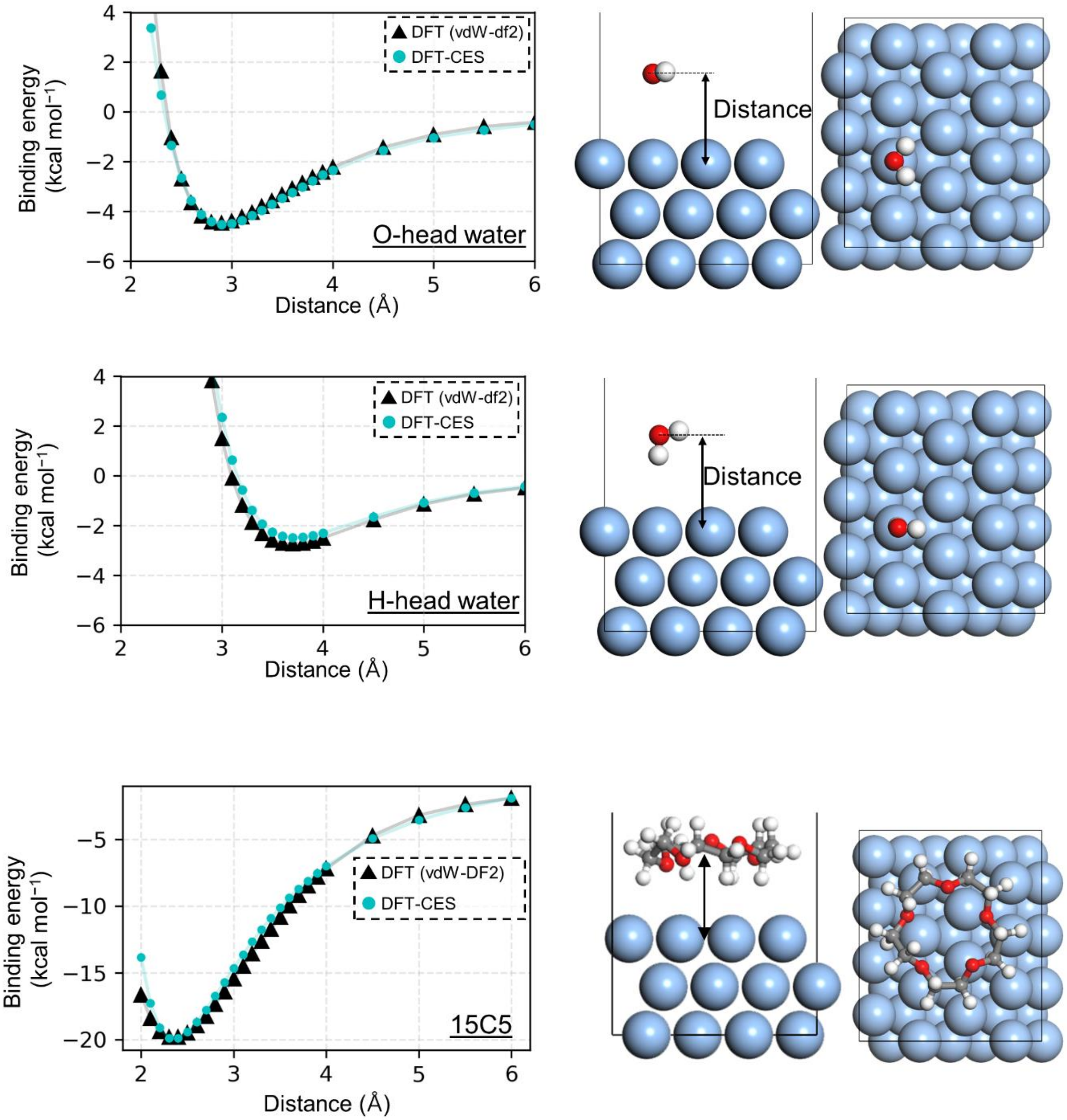

Notably, our previous studies have demonstrated that the current parameterization strategy enables the reliable prediction of the solid–liquid interfacial tension and thereby, the prediction of the experimental contact angle[12].

The optimized parameters for Ag-water interaction was further benchmarked using Ag hexamer cluster model. Either when the Ag hexamer is neutral or −1 charged, DFT-CES reproduces the QM-level water binding energy curves to the Ag hexamer both for O-head and H-head orientations. Here, we employed the Grimme's D3 vdW correction method as coupled with HSE06 functional using NWChem 6.8 software[13]. We employed an effective core potential for the core electrons of the Ag atom and def2-TZVP basis set[14].

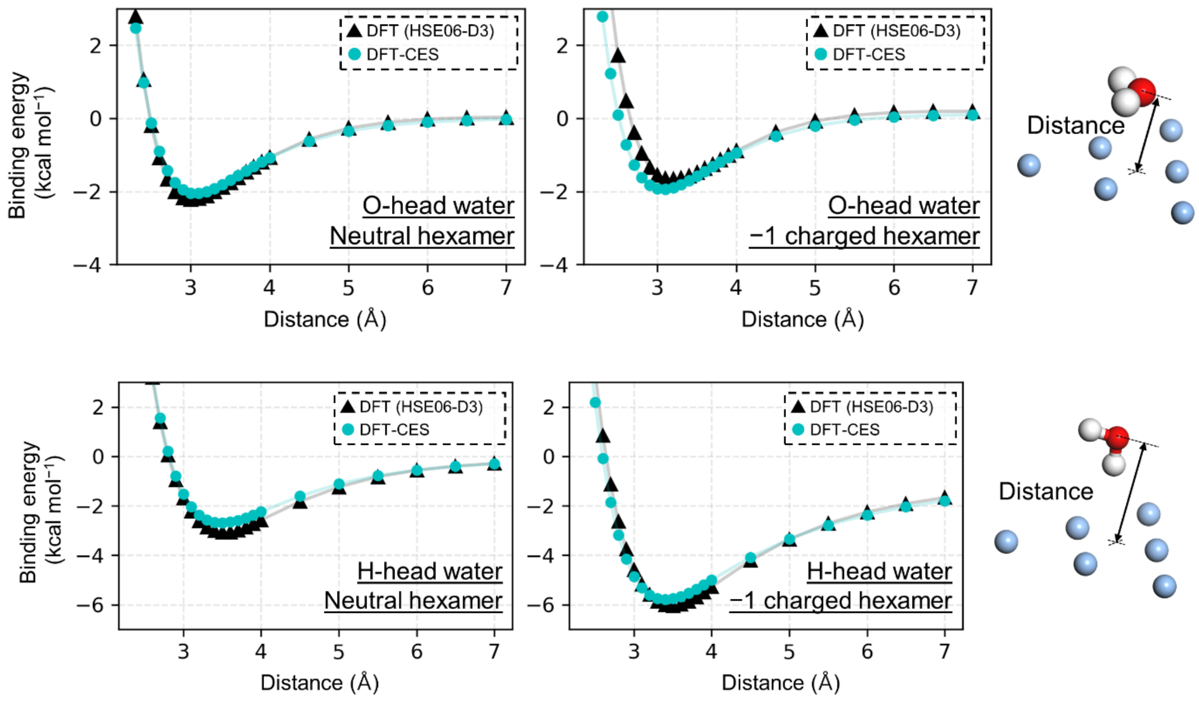

We developed FF parameters to model the Ag–Na$^+$ and Ag–F$^-$ interactions. Because Na$^+$ and F$^-$ are monoatomic ions, their atomic $C_6$ parameters can be unambiguously determined based on the Casimir–Polder integral of the dipole polarizability through the time-dependent DFT calculations[15]. In addition, using the $C_6$ parameter of Ag calculated in the same manner, we determined the off-diagonal $C_{6,ij}$ terms for the Ag–Na$^+$ pair, and the Ag–F$^-$ pair was determined by following the combination rule suggested in the reference[15].

To determine the $A$ and $R$ parameters that are attributed to the Pauli repulsion energy, we obtained binding energy curves of Na$^+$ and F$^-$ to the Ag hexamer, through Hartree-Fock calculations. Because of the existence of a net charge in the system, we avoided employing a slab model; instead, we chose the Ag hexamer having a planar structure with a closed singlet state. We also note that the Hartree–Fock energy completely embodies the contribution of the Pauli exchange interaction with no correlation (which originates the dispersion) energy, and therefore, it is ideal to selectively model the Pauli repulsion energy. Hartree–Fock calculations were performed using the NWChem 6.8 software[13] by employing an effective core potential for the core electrons of the Ag atom and large-sized Gaussian basis sets with minimal basis set incompleteness error (def2-QZVPPD)[14].

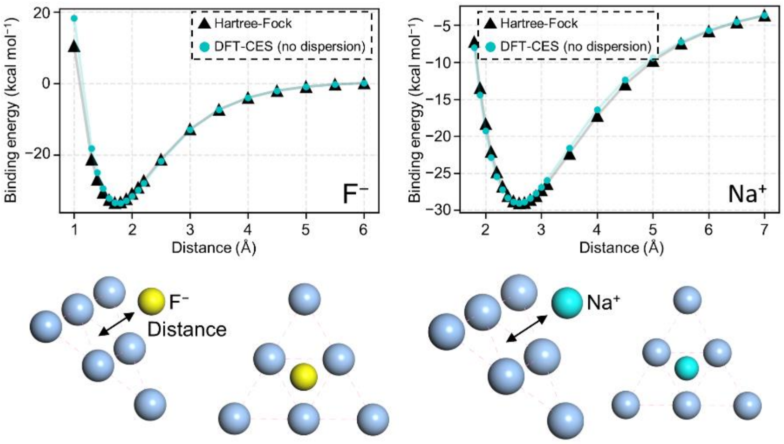

| | $A$ (kcal mol$^{-1}$) | $R$ (Å) | $C_6$ (kcal mol$^{-1}$ Å$^6$) |
|---|---|---|---|
| Ag–$H_{water}$ | 3389 | 0.343 | 355 |
| Ag–$O_{water}$ | 17427 | 0.348 | 1739 |
| Ag–$C_{15C5}$ | 129999 | 0.314 | 1500 |
| Ag–$H_{15C5}$ | 99999 | 0.241 | 240 |
| Ag–$O_{15C5}$ | 126295 | 0.286 | 884 |
| Ag–$Na^+$ | 6700 | 0.450 | 174 |
| Ag–$F^-$ | 88040 | 0.260 | 2049 |

Supplementary Note #5. Calculation of absolute electrode potential, $E^{(\mathrm{abs})}$

We developed a DFT-CES simulation cell consisting of a metal electrode–electrolyte interface that is periodic along the $x$-direction and $y$-direction and aperiodic in the $z$-direction; therefore, it includes an additional vacuum region along the $z$-direction. Considering the $z$-directional aperiodicity that is intrinsic to the interfacial system, a sufficiently long scale is required to appropriately model the entire potential drop across the electric double layer (EDL) based on the DFT-CES approach.

Because of the QM description of the electrons in the metal electrode using DFT, the potential at the Fermi level of the metal electrons, $E_{\mathrm{F}}$, is well-defined and can be referenced with respect to the flat potential in the vacuum region, $\phi_{\mathrm{v}}$, by computing the electrostatic potential of the overall metal–electrolyte–vacuum system (**Supplementary Fig. 1**). The potential difference between $E_{\mathrm{F}}$ and $\phi_{\mathrm{v}}$ corresponds to the electric work required to remove a unit charge from the metal to the vacuum across the electrolyte layer, $E^{(\mathrm{abs})}$, according to the definition proposed by Trasatti[16]. Because the absolute potential of the standard hydrogen electrode (SHE) is estimated to be between 4.4 and 4.8 V[16], that is set as 4.4 V in this study[17], the electrode potential, $E$, relative to the SHE, becomes,

$$E\ (\text{vs SHE}) = E^{(\mathrm{abs})} - 4.4 = \phi_{\mathrm{v}} - E_{\mathrm{F}} - 4.4.$$

We calculated $E$ at the point of zero charge ($E_{\mathrm{PZC}}$) by interfacing various (zero-charged) metal surfaces with salt-free water slabs. The theoretical values are consistent with the available experimental data (**Supplementary Fig. 2**) that implies that our simulation accurately models the potential difference across the interface.

Supplementary Note #6. Analogical mapping of EDL onto two-plate capacitor model

For an intuitive understanding of the EDL capacitance behavior, it is useful to map the EDL onto a simple two-plate capacitor model that is defined based on the charge-separation distance, $d$, and effective dielectric constant, $\varepsilon_{\mathrm{eff}}$. This analogical analysis also provides a conceptual link between our simulation data and that of the classical EDL theory that considers the EDL as two oppositely charged layers of charged electrode and counter ions, and water is often simplified as a field-screening medium.

$d$ is defined as the mean separation distance between the excess charge accumulated in the electrode and the counter-ion charge in the electrolyte along the $z$-direction (surface normal).

$$d = \frac{\int_0^\infty z\rho_{\mathrm{electrode}} dz}{\int_0^\infty \rho_{\mathrm{electrode}} dz} - \frac{\int_0^\infty z\rho_{\mathrm{ion}} dz}{\int_0^\infty \rho_{\mathrm{ion}} dz}$$

$\varepsilon_{\mathrm{eff}}$ must quantify the average field-screening ability of the water dipoles in the EDL. Because the overall potential drop across the interface, $\phi_{\mathrm{d}}$, should be the same for both the actual EDL and the two-plate capacitor model for the same $E$, $\varepsilon_{\mathrm{eff}}$ is defined as

$$\varepsilon_{\mathrm{eff}} = \frac{\sigma d}{\varepsilon_0 \phi_d},$$

where $\sigma$ is the surface charge density, and $\varepsilon_0 = 8.85 \times 10^{-12}$ F m$^{-1}$ is the vacuum permittivity; $\phi_{\mathrm{d}}$ is calculated from our simulation results.

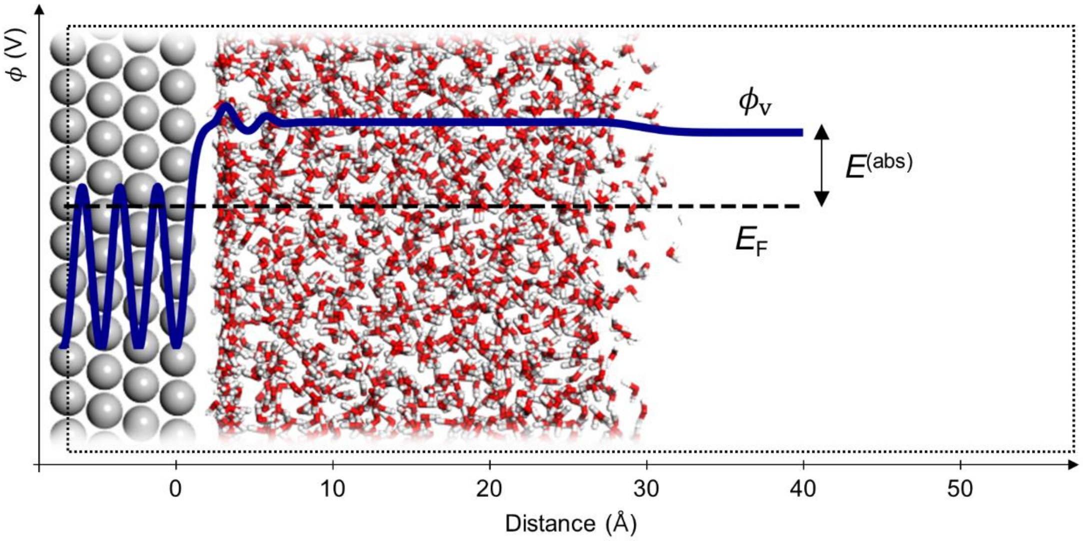


**Supplementary Fig. 1. Definition of an absolute electrode potential, $\boldsymbol{E^{(\mathrm{abs})}}$.** An electrostatic potential profile, $\phi$, along the $z$-direction is plotted above a snapshot at the point of zero charge (PZC), where $E_{\mathrm{F}}$ and $\phi_{\mathrm{v}}$ are the Fermi level and vacuum potential, respectively. $E^{(\mathrm{abs})}$ is defined based on the difference between $E_{\mathrm{F}}$ and $\phi_{\mathrm{v}}$.

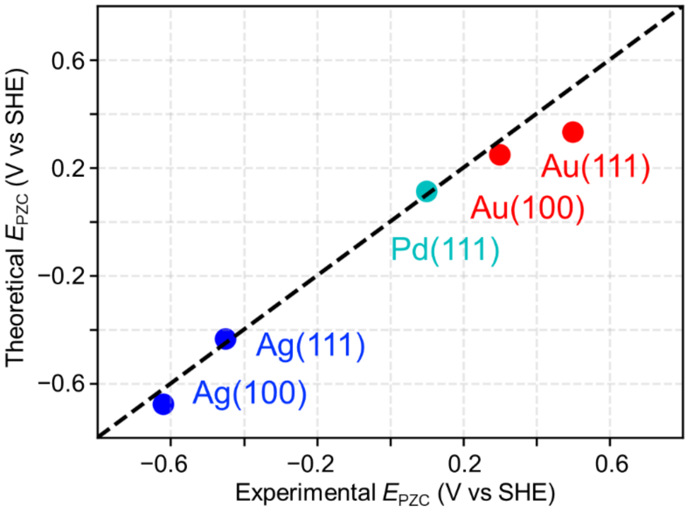


**Supplementary Fig. 2. Theory–experiment comparison of potential at the point of zero charge, $E_{PZC}$.** $E_{PZC}$ of various single-crystalline surfaces is calculated based on the density functional theory in classical explicit solvents (DFT-CES) simulation ($y$-axis) that is compared with the experimental data of the previous studies[18–21].

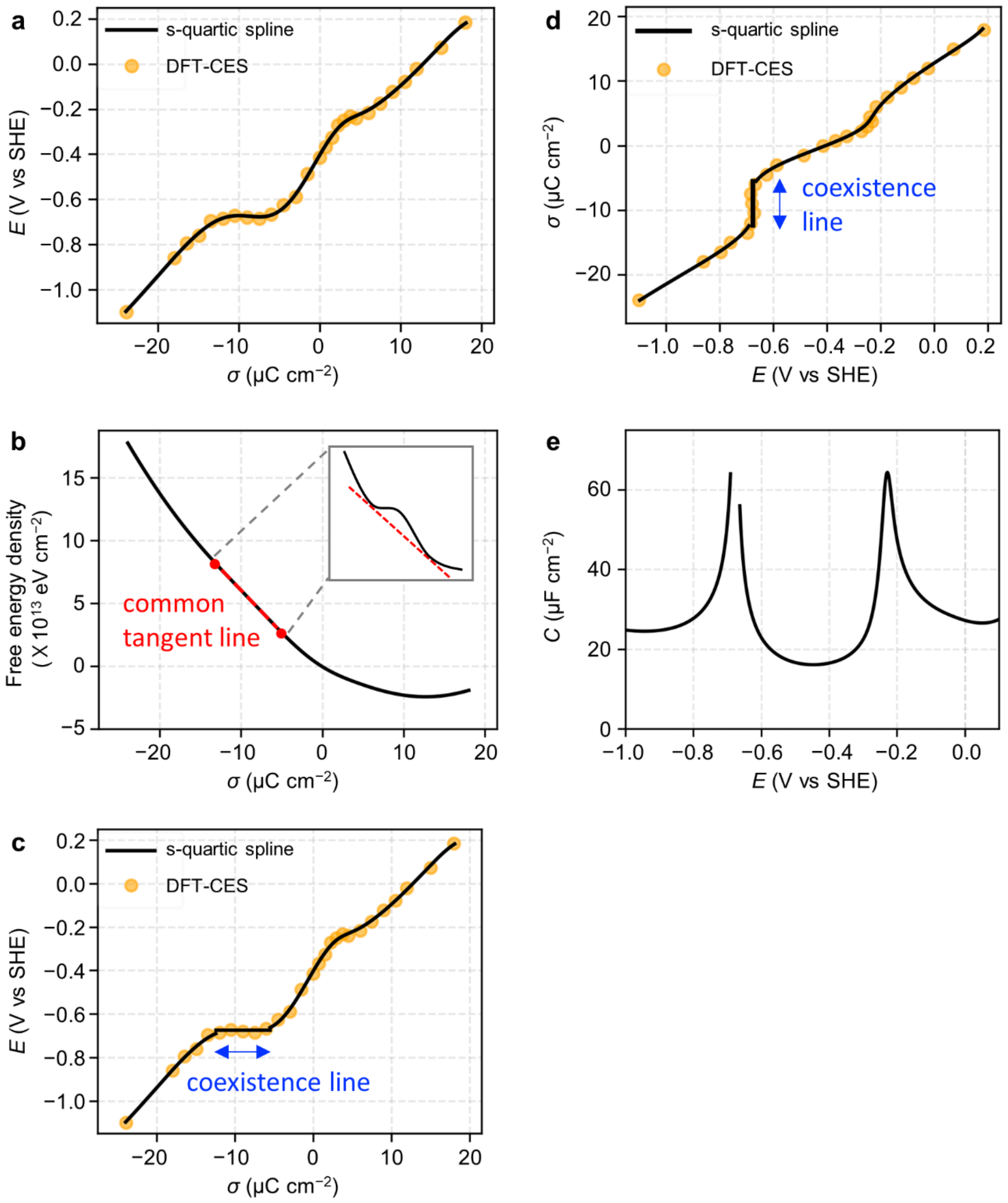


**Supplementary Fig. 3. Construction of Maxwell tie-line.** Numerical integration of electrode potential, $E$, with respect to surface charge density, $\sigma$, yields the free energy profile (**a** $\rightarrow$ **b**), where double minima appear (inset of **b**). The density functional theory in classical explicit solvents (DFT-CES) data are fitted by smoothed quartic (s-quartic) spline function. A common

tangent line of two minima can be obtained, yielding a constant $E$ in the corresponding $\sigma$ range in the $E$-$\sigma$ curve (**b** → **c**). By reversing the axes, $\sigma$-$E$ curve is obtained, where the coexistence line becomes a vertical line (**c** → **d**), and the differentiation of $\sigma$ with respect to $E$ yields the differential capacitance, that is, the $C$-$E$ curve (**d** → **e**).

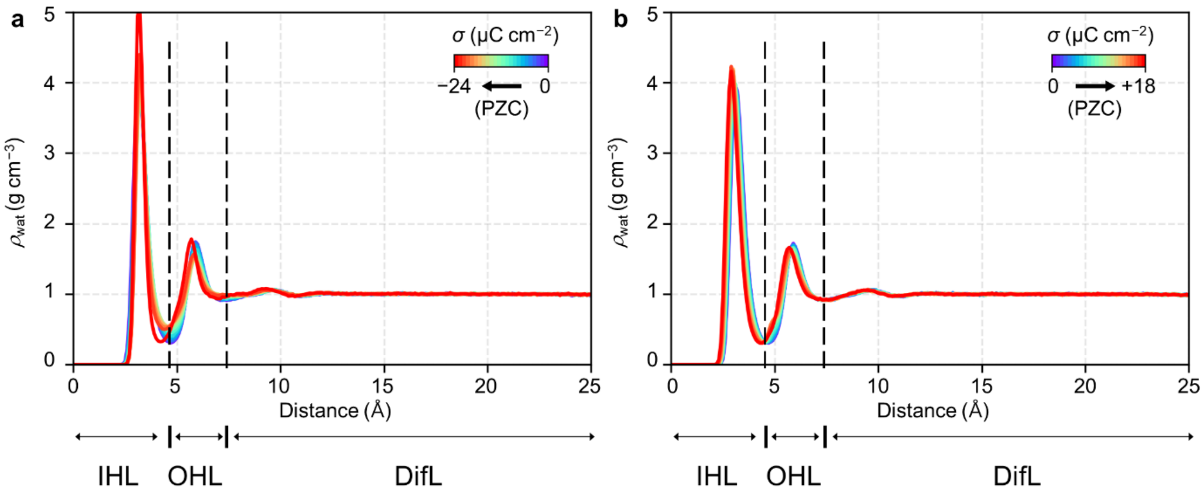


**Supplementary Fig. 4. Local water density, $\rho_{\mathrm{wat}}$, profiles along the surface normal.** $z$-directional $\rho_{\mathrm{wat}}$ when interfaced with the **a,** negatively charged electrode (surface charge density, $\sigma < 0$), and **b,** positively charged electrode ($\sigma > 0$). As the electrode is charged further from the point of zero charge (PZC), the color-code changes from violet to red. The location of the inner Helmholtz layer (IHL), outer Helmholtz layer (OHL), and diffuse layer (DifL) are defined based on $\rho_{\mathrm{wat}}$.

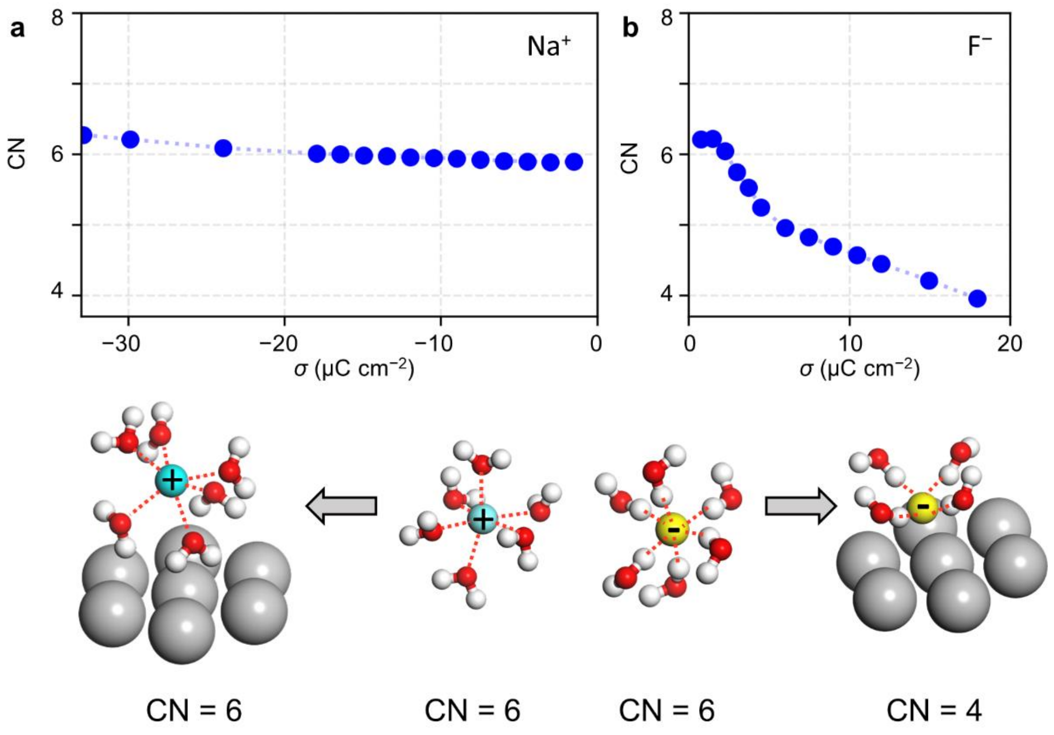


**Supplementary Fig. 5. Change of ion-water coordination number (CN).** The CN of **a,** $Na^{+}$ (near the negatively charged electrode) and **b,** $F^{-}$ (near the positively charged electrode), upon changing the surface charge density, $\sigma$, are shown. A cutoff distance from the center of the ion defining the CN is set to 3.2 Å. The scheme in the lower row shows the change in the water coordination structure of the ions.

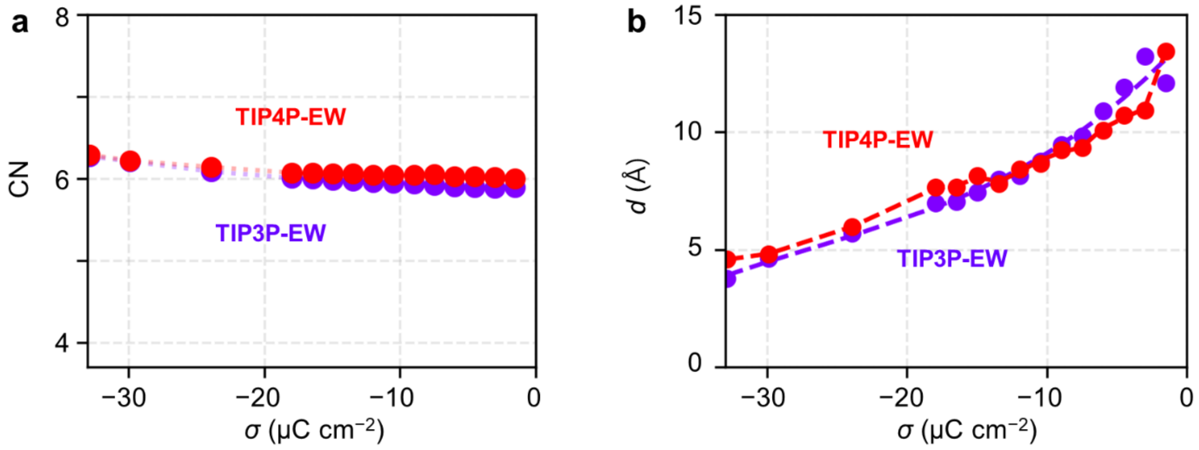


**Supplementary Fig. 6. Density functional theory in classical explicit solvents (DFT-CES) results for negatively charged electrode employing TIP4P-EW water model. a,** Coordination number (CN) as a function of surface charge density, $\sigma$ and **b,** charge-separation distance, *d*, as a function of $\sigma$, using two different water models of TIP3P-EW (violet) and TIP4P-EW (red).

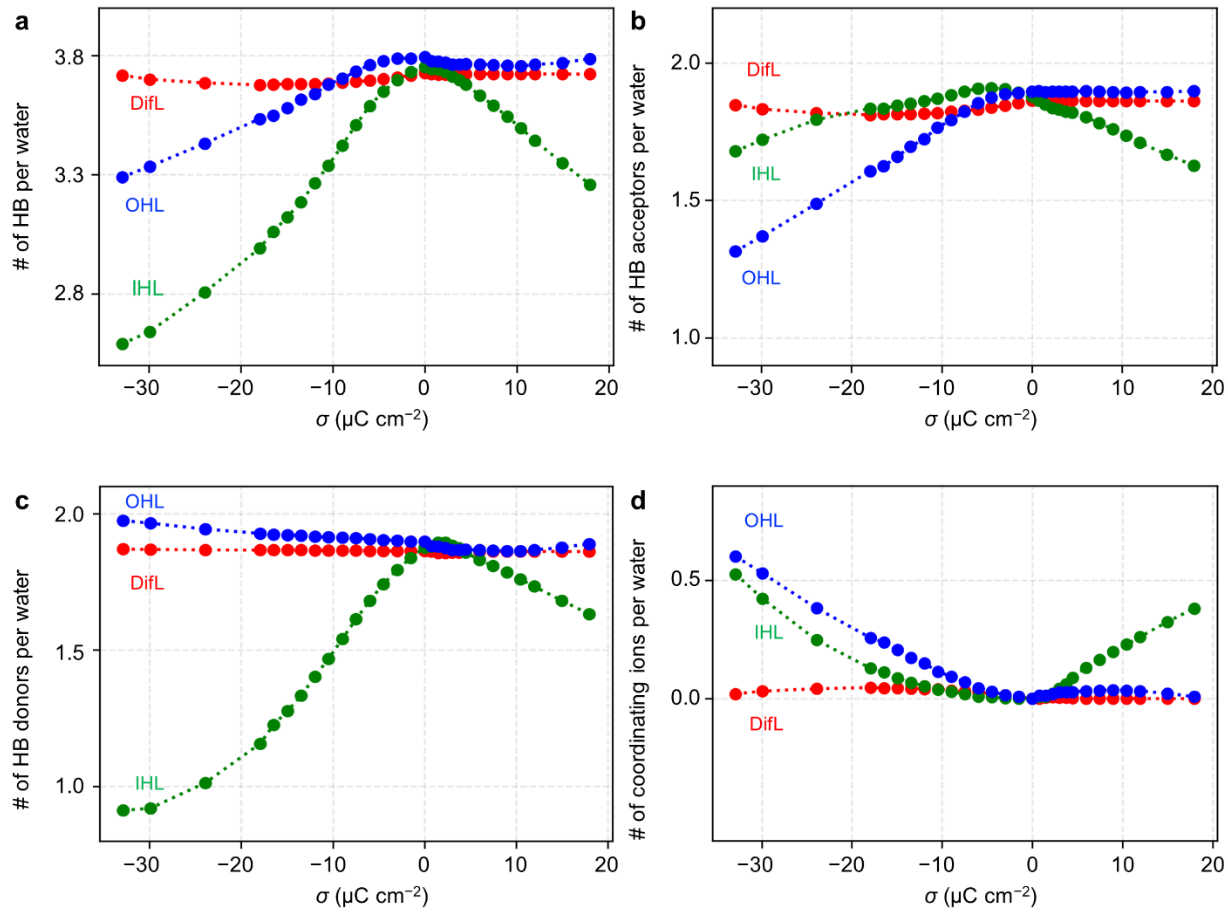


**Supplementary Fig. 7. Layer-resolved hydrogen bond (HB) network analysis. a,** Total number, #, of HB per water molecule as a function of the surface charge density, $\sigma$, that is expressed as a sum of the **b,** # of HB acceptors and **c,** # of HB donors per water molecule in the inner Helmholtz layer (IHL), outer Helmholtz layer OHL, and diffuse layer (DifL). **d,** # of coordinating ions per water molecule in each layer is also shown. When the electrode is positively charged ($\sigma > 0$), the # of HB donors and acceptors in the IHL simultaneously decreases, while the # of coordinating ions per water molecule increases only in the IHL. This implies that the HB network formed within the IHL collapses, owing to the adsorbed ions. When the electrode is negatively charged ($\sigma < 0$), the # of HB donors in the IHL and the # of HB acceptors in the OHL predominantly decreases, while the # of coordinating ions per water

molecule increases both in IHL and OHL. This implies that the HB network formed across the two layers of IHL and OHL collapses due to the accumulation of ions in the OHL.

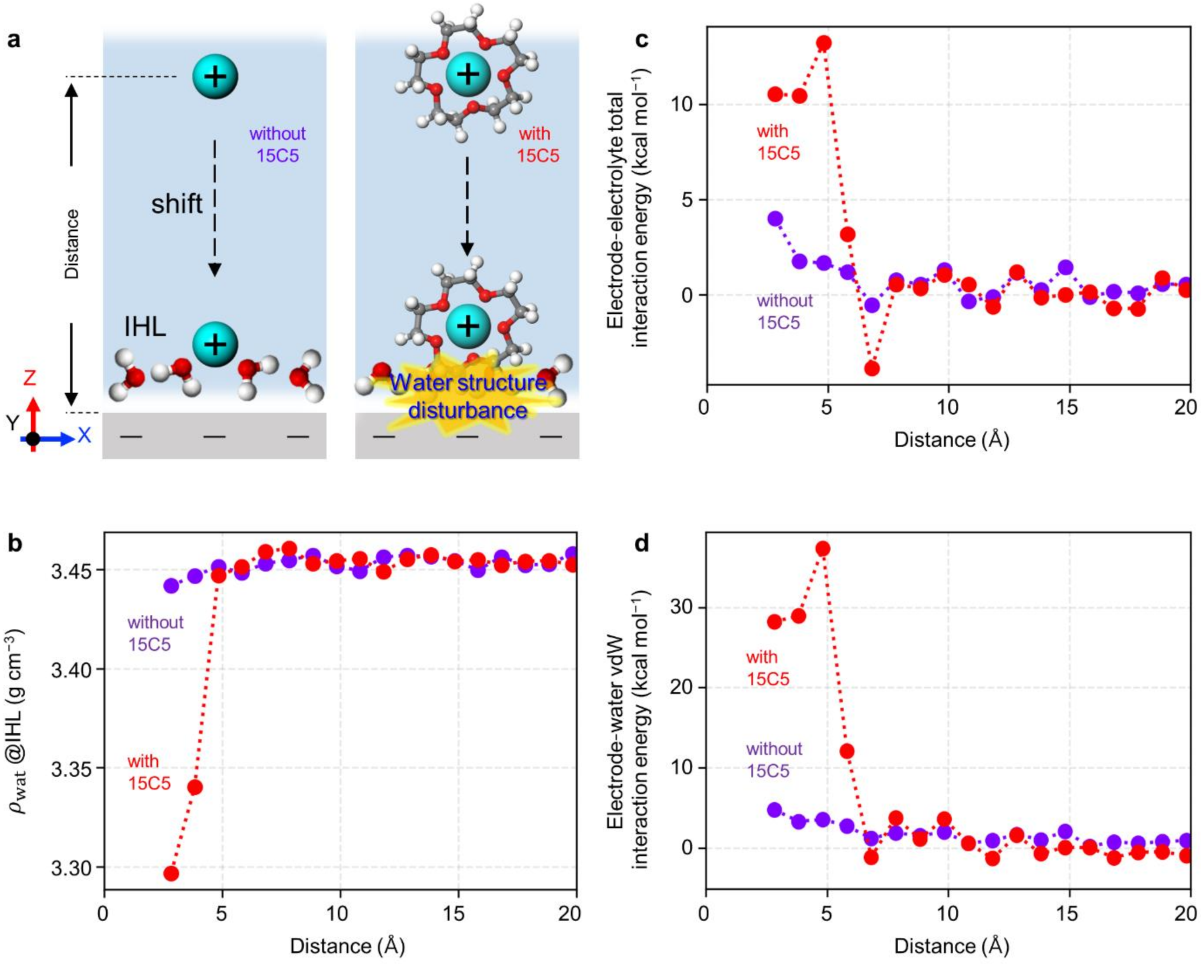


**Supplementary Fig. 8. Origin of uncondensed EDL structure formation following the 15-Crown-5 (15C5) complexation of cation. a,** To trace the origin of the increase in the charge-separation distance, *d*, additional constrained molecular dynamics (MD) simulations were performed. Two $Na^+$ cations were included in the electrolyte phase that were charge-balanced with the charged electrodes, and the *z*-directional (surface normal) distance of the two cations from the electrode surface was constrained. For each separation distance, 1.5 ns canonical MD simulations were performed, and the last 1 ns trajectory was sampled for analyses. Varying the separation distance, the **b,** local density of water adlayer at the IHL, $\rho_{\mathrm{wat}}$@IHL, **c,** electrode–electrolyte interaction energy, and **d,** electrode–water van der Waals (vdW) energy are compared

whether the cations are complexed (with 15C5) or not (without 15C5). When the 15C5-complexed $Na^+$ approaches the electrode within approximately 5 Å from the electrode, it disturbs the stable water adlayer structure formed at the IHL, causing a substantial increase in the electrode-electrolyte interaction energy that is mostly ascribed to the loss of electrode–water vdW interaction. Such a situation is the opposite of the case of uncomplexed $Na^+$ that can be stably coordinated by the water adlayer at a close distance from the electrode (see the panel **a**).

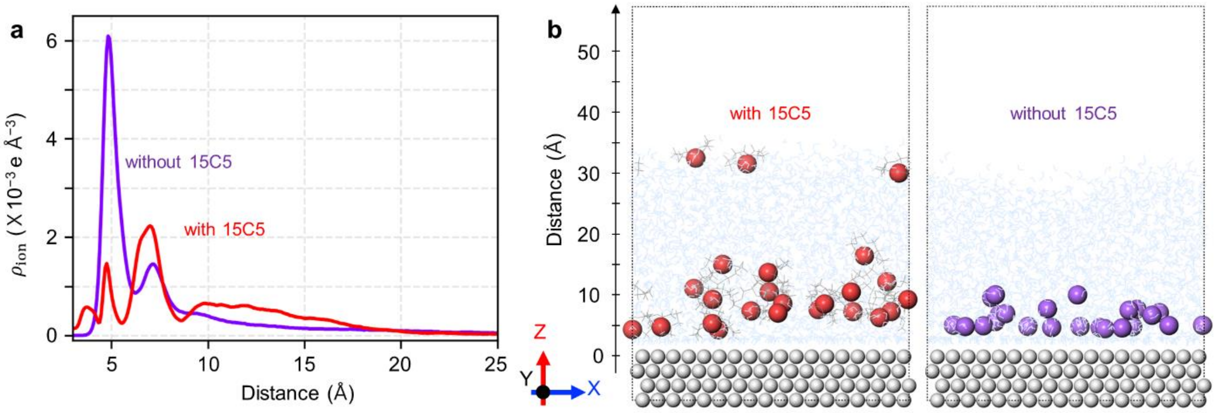


**Supplementary Fig. 9. Local density profiles of $Na^+$, $\rho_{ion}$, along the surface normal. a,** 15-Crown-5 (15C5)-complexed $Na^+$ case (red) is compared with the uncomplexed case (violet) when the electrode is charged at a surface charge density, $\sigma = -18$ μC $cm^{-2}$. **b,** Representative snapshots showing the formation of uncondensed EDL structure for the 15C5-complexed $Na^+$ (left; red spheres denote $Na^+$) and the formation of compact EDL structure for the uncomplexed $Na^+$ (right; violet spheres denote $Na^+$). Grey sticks and cyan sticks represent 15C5 and water, respectively.

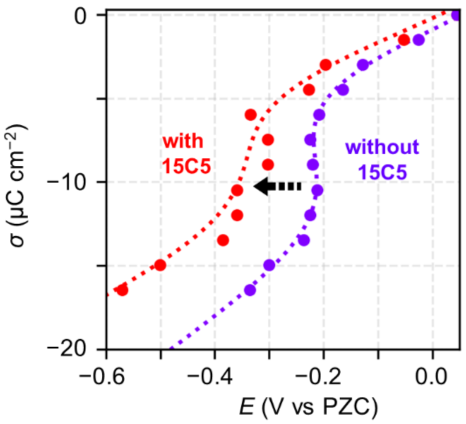


**Supplementary Fig. 10. Shift of the $\sigma$-$E$ curve after cation complexation.** Increase in charge-separation distance, $d$ after the cation complexation with 15-Crown-5 (15C5) shifts the S-shaped region to the more negative potential.

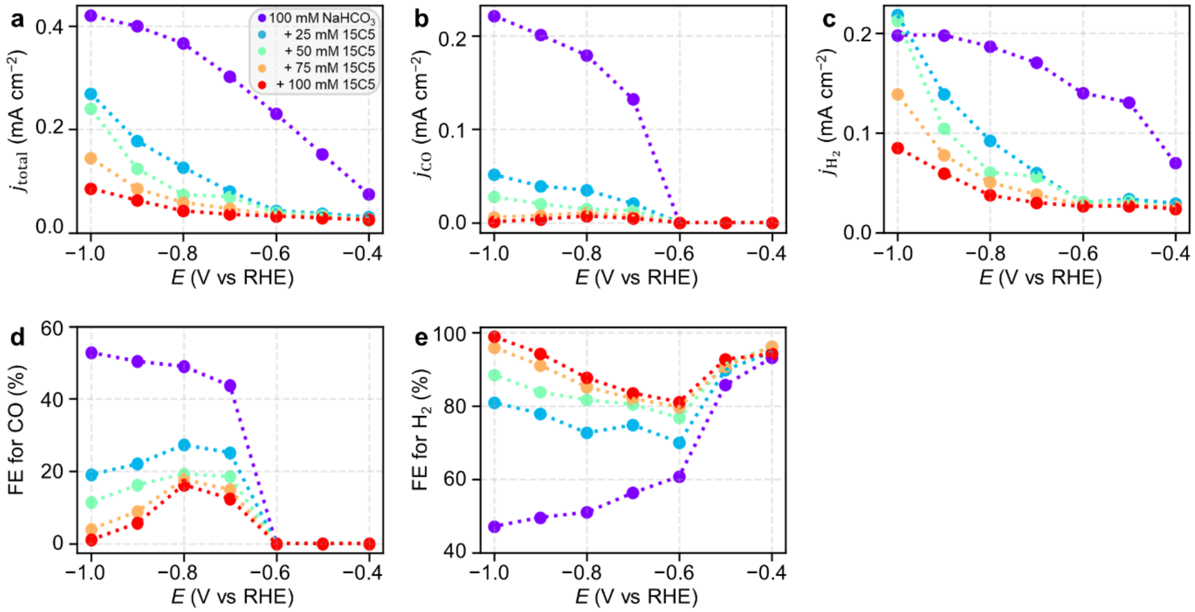


**Supplementary Fig. 11. Electrochemical measurements on Ag(111) for carbon dioxide reduction reaction ($CO_2$RR) in the experiments. a,** Total current density, $j_{\mathrm{total}}$. **b,** Partial current density for carbon monoxide, $j_{\mathrm{CO}}$. **c,** Partial current density for hydrogen, $j_{\mathrm{H_2}}$. **d,** Faradaic efficiency (FE) for CO. **e,** FE for $H_2$. The $CO_2$ electrolysis was performed in a $CO_2$-bubbled 100 mM $NaHCO_3$ electrolyte with different 15-Crown-5 (15C5) concentrations in the range of 0–100 mM.

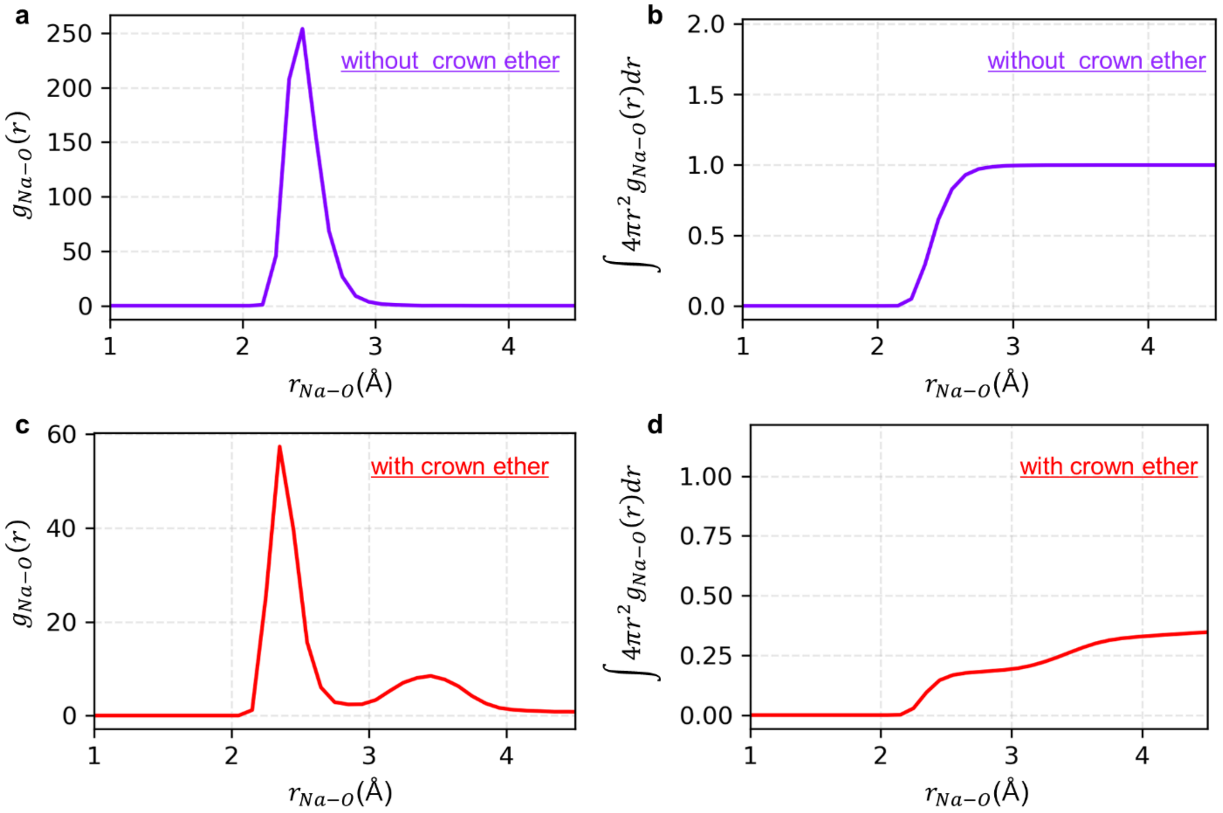


**Supplementary Fig. 12. Radial distribution function, $\boldsymbol{g_{Na-O}(r)}$ and its integrated value, $\boldsymbol{\int 4\pi r^2 g_{Na-O}(r)dr}$ as a function of a distance between $Na^+$ and O of adsorbed $CO_2$. a, b,** $g_{Na-O}(r)$ for uncomplexed $Na^+$ **(a)** and value of $\int 4\pi r^2 g_{Na-O}(r)dr$ **(b)**. **c, d,** $g_{Na-O}(r)$ for 15C5-complexed $Na^+$ **(c)** and value of $\int 4\pi r^2 g_{Na-O}(r)dr$ **(d)**.

## Supplementary References


1. Blöchl, P. E. Projector augmented-wave method. *Phys. Rev. B* **50**, 17953–17979 (1994).
2. Perdew, J. P., Burke, K. & Ernzerhof, M. Generalized gradient approximation made simple. *Phys. Rev. Lett.* **77**, 3865–3868 (1996).
3. Price, D. J. & Brooks, C. L. A modified TIP3P water potential for simulation with Ewald summation. *J. Chem. Phys.* **121**, 10096–10103 (2004).
4. Nosé, S. A unified formulation of the constant temperature molecular dynamics methods. *J. Chem. Phys.* **81**, 511–519 (1984).
5. Hoover, W. G. Canonical dynamics: equilibrium phase-space distributions. *Phys. Rev. A* **31**, 1695–1697 (1985).
6. Yeh, I.-C. & Berkowitz, M. L. Ewald summation for systems with slab geometry. *J. Chem. Phys.* **111**, 3155–3162 (1999).
7. Fyta, M. & Netz, R. R. Ionic force field optimization based on single-ion and ion-pair solvation properties: Going beyond standard mixing rules. *J. Chem. Phys.* **136**, 124103 (2012).
8. Marcus, Y. Thermodynamics of solvation of ions. Part 5.–Gibbs free energy of hydration at 298.15 K. *J. Chem. Soc., Faraday Trans.* **87**, 2995–2999 (1991).
9. Mayo, S. L., Olafson, B. D. & Goddard, W. A. DREIDING: a generic force field for molecular simulations. *J. Phys. Chem.* **94**, 8897–8909 (1990).
10. Jorgensen, W. L., Maxwell, D. S. & Tirado-Rives, J. Development and testing of the OPLS All-Atom force field on conformational energetics and properties of organic liquids. *J. Am. Chem. Soc.* **118**, 11225–11236 (1996).
11. Lee, K., Murray, É. D., Kong, L., Lundqvist, B. I. & Langreth, D. C. Higher-accuracy van der Waals density functional. *Phys. Rev. B* **82**, 081101 (2010).
12. Gim, S., Lim, H.-K. & Kim, H. Multiscale simulation method for quantitative prediction of surface wettability at the atomistic level. *J. Phys. Chem. Lett.* **9**, 1750–1758 (2018).
13. Valiev, M. *et al.* NWChem: A comprehensive and scalable open-source solution for large scale molecular simulations. *Comput. Phys. Commun.* **181**, 1477–1489 (2010).
14. Rappoport, D. & Furche, F. Property-optimized Gaussian basis sets for molecular response calculations. *J. Chem. Phys.* **133**, 134105 (2010).

15. Gould, T. & Bučko, T. $C_6$ coefficients and dipole polarizabilities for all atoms and many ions in rows 1–6 of the periodic table. *J. Chem. Theory Comput.* **12**, 3603–3613 (2016).

16. Trasatti, S. The absolute electrode potential: an explanatory note: (recommendations 1986). *Pure & Appl. Chem.* **58**, 955–966 (1986).

17. Le, J., Iannuzzi, M., Cuesta, A. & Cheng, J. Determining potentials of zero charge of metal electrodes versus the standard hydrogen electrode from density-functional-theory-based molecular dynamics. *Phys. Rev. Lett.* **119**, 016801 (2017).

18. Pajkossy, T., Wandlowski, T. & Kolb, D. M. Impedance aspects of anion adsorption on gold single crystal electrodes. *J. Electroanal. Chem.* **414**, 209–220 (1996).

19. El-Aziz, A. M., Kibler, L. A. & Kolb, D. M. The potentials of zero charge of Pd(111) and thin Pd overlayers on Au(111). *Electrochem. commun.* **4**, 535–539 (2002).

20. Valette, G. Double layer on silver single-crystal electrodes in contact with electrolytes having anions which present a slight specific adsorption Part II. The (100) face. *J. Electroanal. Chem.* **138**, 37–54 (1982).

21. Valette, G. Double layer on silver single-crystal electrodes in contact with electrolytes having anions which present a slight specific adsorption Part III. The (111) face. *J. Electroanal. Chem.* **269**, 191–203 (1989).
